# Effect of Surface Wettability and Energy on Bacterial Adhesion to Dental Aligners: A Comparative In Vitro Study

**DOI:** 10.3390/bioengineering12090898

**Published:** 2025-08-22

**Authors:** A. Martínez Gil-Ortega, M. M. Paz-Cortés, M. J. Viñas, P. Cintora-López, A. Martín-Vacas, J. Gil, J. M. Aragoneses

**Affiliations:** 1 Faculty of Dentistry, Alfonso X El Sabio University, 28691 Madrid, Spain; anagil@uax.es (A.M.G.-O.); mpazcor@uax.es (M.M.P.-C.); mvinapin@uax.es (M.J.V.); pcintlop@uax.es (P.C.-L.); jmaragoneses@gmail.com (J.M.A.); 2 Bioinspired Oral Biomaterials and Interfaces, Department Ciencia e Ingeniería de Materiales, Escola d’Engenyeria Barcelona Est, Universitat Politècnica de Catalunya, Av. Edurad Maristany 16, 08019 Barcelona, Spain; 3 Department of Dental Research, Federico Henriquez y Carvajal University, Santo Domingo 10106, Dominican Republic

**Keywords:** wettability, bacteria, surface free energy, orthodontic aligner, polymers, polyurethanes

## Abstract

The use of orthodontic aligners has increased significantly due to their convenience and esthetic advantages. However, understanding their microbiological behavior and cytotoxicity is essential. This study aimed to evaluate the metabolic activity (MA) and proliferation of different bacterial strains—assessed through colony-forming unit (CFU) counts—as well as the cytotoxicity of three widely used aligner systems: Spark, Invisalign, and Smile. Wettability and surface free energy (both dispersive and polar components) were determined using the sessile drop technique. The bacterial strains *Streptococcus oralis*, *Actinomyces viscosus*, *Streptococcus gordonii*, *Enterococcus faecalis*, and *Porphyromonas gingivalis* were cultured, and their behavior on the aligner surfaces was assessed under simulated oral cavity conditions in both aerobic and anaerobic environments using a bioreactor. Cytocompatibility was evaluated with HFF-1 human fibroblasts. Distinct strain-specific behaviors were observed. For Spark aligners, the contact angle was 70.5°, Invisalign 80.6°, and Smile 91.2°, and the surface free energy was 60.8, 66.7, and 74. 2 mJ/m^2^, respectively, highlighting the high polar component of the Spark aligner of 31.9 mJ/m^2^ compared to 19.3 and 20.2 mJ/m^2^ for Invisalign and Smile, respectively. The Spark aligner exhibited the lowest metabolic activity for *Streptococcus oralis* (23.1%), *Actinomyces viscosus* (43.2%), *Porphyromonas gingivalis* (17.7%), and biofilm formation (2.4%), likely due to its higher hydrophilicity. The Smile aligner showed the lowest metabolic activity for *Streptococcus gordonii* (23.6%) and *Enterococcus faecalis* (51.1%), attributed to its low polar surface free energy component. CFU counts were minimal for all aligners and bacterial strains, including biofilm. All aligners demonstrated cytocompatibility above 70% (Spark: 71.0%, Invisalign: 75.7%, and Smile: 75.6%). These findings highlight the importance of considering aligner material properties in clinical practice and underscore the need for proper oral hygiene and aligner maintenance.

## 1. Introduction

The oral cavity constitutes a highly dynamic and complex ecosystem that, under physiological conditions, harbors a diverse microbiota composed of multiple bacterial species that confer benefits to the host. This balance, however, can be disrupted, leading to the proliferation of pathogenic species and the onset of oral diseases such as dental caries—frequently associated with *Streptococcus mutans*—and periodontal conditions including gingivitis and periodontitis, often linked to *Porphyromonas gingivalis*. Beyond its role in oral health, the oral microbiome contributes to immune regulation and provides resistance to pathogenic colonization [1].

Orthodontic treatment can induce inflammatory responses in periodontal tissues, potentially worsening pre-existing gingival conditions and accelerating progression toward periodontitis. This inflammation is associated with increased levels of interleukin-6 (IL-6), a cytokine upregulated during periodontal disease as part of the host response to bacterial challenge [2]. Numerous studies have documented early periodontal deterioration during orthodontic treatment, particularly within the first months, primarily due to increased plaque retention and the associated challenges in maintaining optimal oral hygiene [3,4]. According to the most recent systematic review on the global prevalence of dental caries in children, Huang et al. (2024) reported a 15.25% increase in incident cases of caries in permanent teeth among children aged 5–14 years between 1990 and 2019, with incidence rates remaining relatively stable over this period (34.04% in 1990 and 33.93% in 2019) [5].

In recent years, clear aligners have become increasingly popular in orthodontic therapy for both pediatric and adult patients. Their appeal lies in the perception that they facilitate better oral hygiene compared to fixed appliances. However, while aligners may appear visually clean, this perception can be misleading. Evidence indicates that children wearing conventional removable appliances exhibit a higher risk of developing caries compared with untreated peers [6]. Moreover, enamel demineralization and the development of white spot lesions have been reported in patients treated with clear aligners [7].

The majority of existing research on clear aligners focuses on adult populations, often comparing periodontal outcomes with those of patients treated with conventional multi-bracket appliances [8,9]. Understanding the material composition of aligners is essential, as their mechanical behavior and clinical performance are directly influenced by the type and properties of the polymers used. Commonly employed materials include polyester-based polymers (polyethylene terephthalate [PET] and polyethylene terephthalate glycol-modified [PETG]), polyurethane, polypropylene, polycarbonate, ethylene–vinyl acetate, and polyvinyl chloride [10,11]. Modern aligners are often composed of polymer blends to optimize mechanical, chemical, and optical characteristics. While the general composition of these materials is known, commercial manufacturers do not disclose precise formulations. Additionally, aligner thickness—ranging from 0.5 mm to 1.5 mm—affects key physical properties, including water absorption, tensile strength, tear resistance, elongation at break, and stress relaxation [12].

Once in use, aligners are exposed to the oral environment, favoring bacterial plaque accumulation, biofilm adhesion, and alterations in color and transparency. These changes are influenced by factors such as surface roughness and porosity [13,14]. Even prior to intraoral use, aligners may exhibit inherent surface irregularities, which tend to worsen over time [15]. Their performance is also shaped by the thermoforming process used during manufacturing, which imposes both thermal and mechanical stresses on the material. Ryokawa reported that thermoforming results in variable thickness reduction, with copolyesters (Essix A) and polycarbonates being less dimensionally stable than polypropylene and PETG [16,17]. Staderini further noted that the effects of thermoforming on surface roughness remain underexplored; increased roughness in PETG aligners, combined with inadequate hygiene, promotes bacterial colonization and discoloration through absorption of saliva, water, and dietary pigments [16].

**Figure 1:**
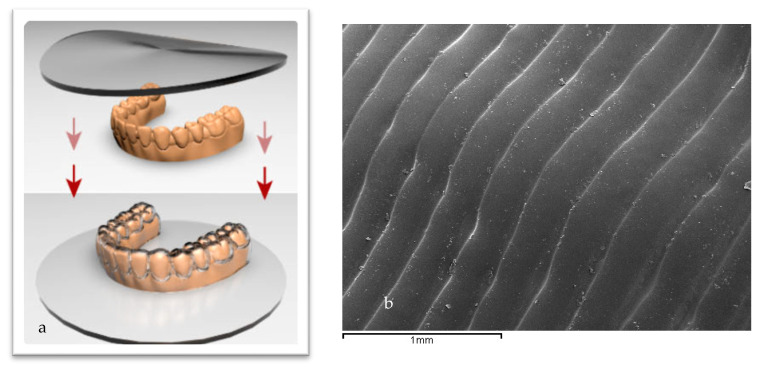
(**a**). Scheme of the process of fabrication of aligners. (**b**). Folders on the aligners produced by the plastic deformation in the thermoforming [18,19,20]. The arrows indicate the direction of stamping in the aligner forming process.

Bacterial adhesion is influenced not only by the intrinsic material properties but also by manufacturing techniques. As shown in Figure 1a, aligners are typically fabricated by thermoforming a polymer sheet over a dental model—a process that may introduce structural defects such as folds, porosities, fissures, and voids, which serve as niches for bacterial colonization (Figure 1b) [15,18,19]. Both in vitro and in vivo studies have demonstrated that aligners undergo degradation over time, with reported changes including loss of mechanical strength, development of microcracks, and discoloration. These effects have been well documented for polyurethane-based aligners such as Invisalign and for other materials including PETG, multilayer polymers, and composite systems, particularly when exposed to bacterial biofilm [15,19,20,21].

Bacterial colonization and biofilm formation begin with adhesion to a surface, a process influenced by bacterial properties, environmental conditions, and surface characteristics. Key surface factors include charge density, wettability, roughness, stiffness, and topography [22,23,24]. Wettability, governed by surface free energy and roughness, plays a central role in mediating interactions between solid and liquid phases, and thereby affects bacterial attachment. Various physicochemical models, including thermodynamic, DLVO, and extended-DLVO theories, have been used to explain adhesion based on van der Waals, electrostatic, and acid–base interactions [25,26]. While hydrophobic bacteria tend to adhere to hydrophobic surfaces and hydrophilic bacteria to hydrophilic ones, the findings on moderately hydrophobic/hydrophilic surfaces are inconsistent. Engineered superhydrophobic or superhydrophilic materials can markedly reduce bacterial adhesion, highlighting the potential of surface modification strategies to control biofilm formation [27,28,29].

Given these considerations, this study aimed to evaluate in vitro the influence of wettability and surface free energy of different orthodontic aligners on bacterial adhesion and cytocompatibility. A negative hypothesis of this study could be that wettability and surface free energy do not affect bacterial proliferation behavior, and a positive hypothesis could be that the physical and chemical properties of the aligner surface influence bacterial colonization and are susceptible to whether the bacteria are aerobic or anaerobic.

## 2. Materials and Methods

### 2.1. Samples Preparation

Two-hundred ten different aligners (Invisalign, Spark and SureSmile) were used (seventy for each type of aligner). This number was obtained by sample size calculation. These aligners were cut by diamond disk cutter (Leco 300, St. Jospeh, MI, USA) using room temperature water as a coolant. The dimensions were different dimensions depending on the test. Then, all the samples were sterilized with UV light 30 min each face.

- Spark: Poliureane resin with 2-hydroxyethyl methacrylate and poly(oxy-1,2-ethanediyl), α,α′-[(1-methylethylidene)di-4,1-phenylene]bis[ω-[(2-methyl-1-oxo-2-propen-1-yl)oxy]-nickel and 2-diethylaminoethyl methacrylate—methanol and trifluoro(methanol)boron. Thickness: 0.75 mm. Commercial Manufacturer: Ormco Corporation, Brea, CA, USA
- Invisalign. Material: LD30, a multi-layer aromatic thermoplastic polyurethane from methylene diphenyl diisocyanate and 1,6-hexanediol plus additives. Thickness: 0.75 mm. Commercial Manufacturer: Align Technology Inc., San Jose, CA, USA
- Smile: Plus, C plus polypropylene/ethylene copolymer (>95%), stabilizers (<5%). Thickness: Plus: 0.035 and 0.040 C plus: 0.040 inches/1 mm. Commercial Manufacturer: Dentsply Sirona, York, PA, USA.

### 2.2. Physicochemical of the Surface Characterization

The three aligners were characterized for their wettability (contact angle), and total surface free energy measured by the sessile drop technique. Ten samples measuring 30 mm in length and 20 mm in width were used from each of the three aligners.

The water sessile drop technique was used for the measurement of the contact angle, θ, formed between the water drop and the surface. The greater the contact angle, the lower the wettability and vice versa. For angles less than 10°, the surface is considered superhydrophilic, for angles between 10° and 90° surfaces are hydrophilic and for angles greater than 90°, surfaces are considered hydrophobic. A droplet generation system equipped with a 500 μL Hamilton syringe with micrometric displacement control was used to control the volume (3 μL) and to deposit the droplet. The analysis was performed using a gonyometer with drop profile image capture (Contact Angle System OCA15plus, DataPhysics, Filderstadt, Germany) and analyzed with SCA20 software version 2.0 (DataPhysics, Filderstadt, Germany).

To calculate the surface free energy, the contact angle was measured with two different liquids, water and diiodomethane. The contact angle measurements of diiodomethane were obtained following the same procedure used to measure water contact angles [22]. The surface free energy and its polar (*γ^p^*) and dispersive (*γ^d^*) components were then calculated using the Owens and Wendt equation [17]:

(1) γL1+cosθ=2γLdγSd12+γLpγSp12

where *γ^d^* and *γ^p^* represent the dispersive and polar components of the liquid surface tension (*γ_L_*), respectively. *θ* denotes the contact angle between the liquid (*L*) and the solid (*S*).

### 2.3. Bacteria Culture

The bacterial strains were selected based on various studies by authors [30,31,32,33,34,35] showing the microorganisms most frequently found in orthodontic aligners. The bacterial strains were acquired from ATTC and subjected to NGS standards (ATCC, Manassas, VA, USA). They comprise fully sequenced, characterized, and authenticated ATCC Genuine Cultures^®^ cultures, which were selected based on relevant phenotypic and genotypic attributes, such as Gram staining, GC content, genome size, and spore formation. Each strain was analyzed five times in One Codex, the leading bioinformatics platform for microbial genomics and metagenomics. The concentrations were: 2 × 10^7^ cells/vial ±10^4^. The bacterial strains are delivered frozen/dried and stored at a temperature of 3 °C.

Different inoculums of *Streptococcus oralis* (ATCC 35037), *Actinomyces viscosus* (ATCC 15987), *Streptococcus gordonii* (ATCC 10558), Enterococcus faecalis (ATCC 19433), and *Porphyromona gingivalis* (ATCC 33277) were performed adding 100 µL of each bacterium in 5 mL of brain heart infusion (BHI) or Trypsin Soy Broth (TSB). Then, the falcons were incubated overnight in anaerobic or aerobic conditions at 37 °C for bacterial growth during 24 h. Ten square samples measuring 10 mm on each side were used for each aligner and for each bacteria strain (10 samples × 5 bacteria strain × 3 aligners = 150 samples). The control used was the empty well. Test was carried out following ASTMD5465-16 [36].

Lately, the inoculums were diluted for the different assays, obtaining an optical density of 0.05 for each bacterium. Moreover, 1 mL of bacteria were added for each sample in a 24-well plate and incubated overnight at 37 °C. The bacteria cultures were performed under aerobic or anaerobic conditions, depending on the bacterium.

### 2.4. Biofilm Formation

An in vitro dynamic biofilm model was used with a sterile source of modified BHI medium, which is transferred to the bioreactor containing the bacterial inoculum via a peristaltic pump operating at a constant pressure and at 30 mL/h. These conditions provoke the growth of the bacterial mixture over specific time intervals. This bioreactor maintains the culture medium under the mouth conditions (37 °C, pH 7.2), and an aerobic or anaerobic atmosphere. This device securely holds aligners such that their surfaces are positioned within a flow channel, exposing the bacteria to controlled conditions and facilitating the formation of biofilms on the aligners. To evaluate the dynamic of biofilm formation on aligners surfaces, the incubation times were set at 24 h.

After 24 h, the supernatant of each well was transferred to another 24-well plate and the samples were washed with PBS. Then 1 mL of resazurin was added into each well of the plate containing the samples. Then, the 24-well plate was incubated at 37 °C for 10 min, and then transferred to a black 96-well plate to measure the fluorescence in the spectrophotometer (Infinite 200 Pro M nano+, TECAN, Männedorf, Switzerland) at 560 nm excitation wavelength and at 590 nm emission wavelength.

To quantify the colony formation units, the different samples were transferred in falcon tubes with 3 mL of PBS and vortexed for 5 min. Then, each solution was diluted in different Eppendorf tubes by the serial dilution method between 100 and 105. Lately, 10 µL of each Eppendorf tube was added in duplicates in different agar plates and incubated for 24 h.

### 2.5. SEM Observations

To observe the bacteria adhesion, the scanning electron microscopy was used (JEOL 6400, Tokyo, Japan) using 20 KV. After the culture, the different samples were washed with PBS and transferred into another 24-well plate. Then, 1 mL of 2.5% of glutaraldehyde was added into each well and left it in the fridge for 10–15 min. Lately, the samples were washed with PBS and dehydrated with different ethanol dilutions (50%, 70%, 90% and 100%). Finally, the samples were left in the desiccator.

### 2.6. Evaluation of the Cytocompatibilty

To evaluate the different aligners toxicity, Human foreskin fibroblasts (HFF-1) were purchased from American Type Culture Collection (ATCC) and cultured with Dulbecco’s Modified Eagle’s Medium (DMEM) supplemented with 15% of Fetal Bovine Serum (FBS) and 1% of Penicillin-Streptomycin (P/S) at 37 °C. Ten square samples measuring 10 mm on each side were used for each aligner. A cell concentration 5 × 10^4^ was seeded on the different samples making three repetitions of each condition and a metabolic assay was performed after 3 days. The medium was removed and 1 mL of resazurin was added in each well and incubated for 1 h at 37 °C. Then, 100 µL of each sample were transferred to a black 96-well plate to measure the fluorescence in the spectrophotometer (Infinite 200 Pro M nano+, TECAN, Männedorf, Switzerland) at 560 nm excitation wavelength and at 590 nm emission wavelength.

### 2.7. Statistical Analysis

All results were expressed as mean and standard error except for the bacterial adhesion test results which were expressed as median and standard error. The comparative T.TEST (with the Excel program version 2018 compilation 14332.20771) was carried out between the different groups at 95%, which means that for values of *p* < 0.05, there are significant differences.

Number of samples

30 for wettability + 150 for bacteria cultures + 30 for citocompatibility = 210 samples.

## 3. Results

Table 1 presents the contact angle values and surface free energy, including their dispersive and polar components, for the three aligners evaluated.

**Figure 2:**
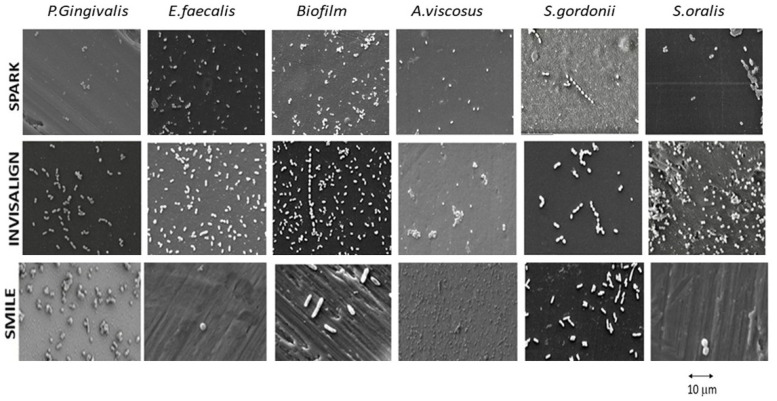
Scanning electron microscope images of the different bacteria strains on the different aligners studied (×1500).

Figure 2 illustrates the bactericidal capacity of the aligners with the different bacterial strains studied.

**Figure 3:**
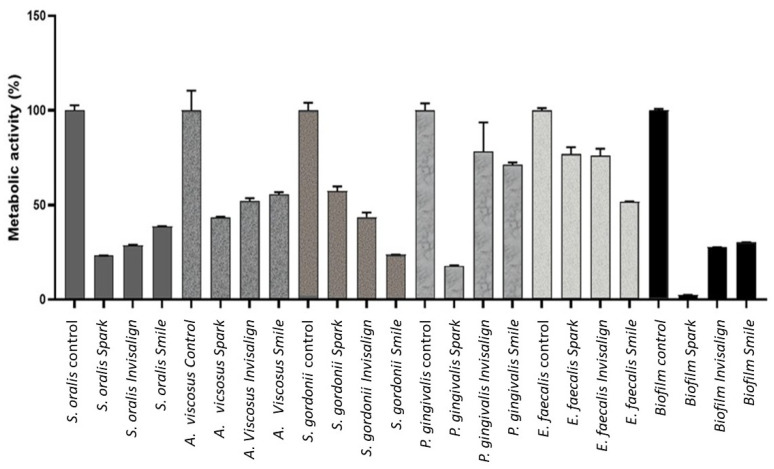
Metabolic activity for different bacteria strains and aligners studied.

Metabolic activity (MA) of the different aligners and bacterial strains is presented in Figure 3, and the results of the statistical analysis are summarized in Table 2. The data revealed statistically significant differences (ANOVA, *p* < 0.0001). Specifically, significant differences were observed for *S. oralis*, *A. viscosus*, *S. gordonii*, *P. gingivalis*, *E. faecalis*, and biofilm formation between the control group and each of the three clear aligners evaluated (*p* < 0.0001 for all. Statistically significant differences were found in *S.oralis*, *A. viscosus*, *S. gordonii*, *P. gingivalis*, *E. faecalis* and biofilm between control and the three clear aligners evaluated (*p* < 0.0001 for all comparisons). Significant differences were also stated between Smile and Spark (*p* < 0.0001) and Suresmile and Invisalign (*p* < 0.05). Related to *A. viscosus*, a significant increase in MA was detected in Spark compared to Suresmile (*p* < 0.05). In relation to *S. gordonii* statistically significant differences were found between all samples (*p* < 0.0001), with the highest MA in Spark and the lowest in Suresmile. Evaluating *P. gingivalis*, a significant lower MA was found in the Spark group compared to Invisalign and Suresmile (*p* < 0.0001 for both comparisons). Analyzing *E. faecalis*, a significant lower MA was found in the Smile groups compared to Spark and Invisalign (*p* < 0.0001 for both comparisons). Statistically significant differences were found between Spark and Invisalign (*p* < 0.0001) and Smile (*p* < 0.0001), with a significant lower MA in the Spark group.

### Quantification of Colony-Forming Units (CFU)

**Figure 4:**
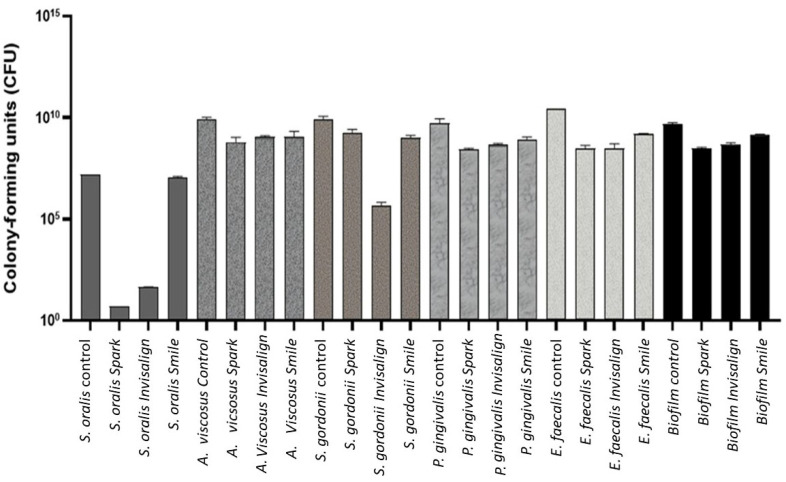
CFU for each bacteria strain for the different aligners studied.

The quantification of colony-forming (CFU) is shown in Figure 4. Statistically significant differences were found (ANOVA *p* < 0.001), and pairwise comparison with post hoc tests were conducted to analyze intergroup differences. Statistically significant higher CFU values were obtained (Table 3) analyzing *A. viscosus*, *S. gordonii* and *E. faecalis* in the control sample compared to all clear aligners. Related to *P. gingivalis*, statistically significant higher CFU values were found in the control sample than Spark and Invisalign.

**Figure 5:**
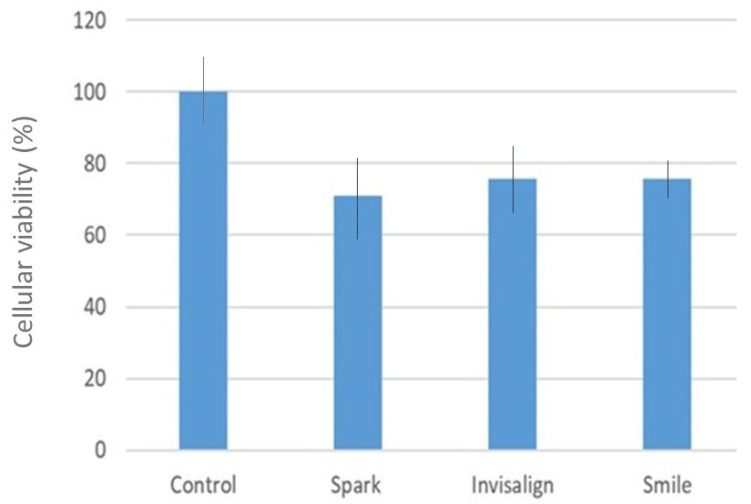
Percentage of cell viability of the different aligners studied.

The percentage of cytocompatibility is shown in Figure 5, while the corresponding statistical analysis is summarized in Table 4. Statistically significant differences were detected in the cytotoxicity evaluation (ANOVA, *p* = 0.0037). Higher cytotoxicity values were observed in the control group compared with Spark (mean difference = 1.351) and Smile (mean difference = 1.525). However, no significant differences were found between the clear aligners.

## 4. Discussion

From the results obtained, we can confirm that the positive hypothesis established is true. The results confirm that different chemical compositions within polyurethanes provide different physical and chemical properties to surfaces that will be in physiological contact with the aligner, which will influence cellular and microbiological behavior. The versatility of the properties of polyurethanes and, in particular, in that of the alienators can be made by the modification of the components that make up the polymer but also by oxygen plasma techniques, application of ultraviolet radiation with ozone, among the most important [37]. In this case, by looking at the chemical compositions we can see the important influence exerted on the wettability properties. It can be seen from the results in Table 1 that there can be differences of 20° in contact angle between Spark and Smile, i.e., the Smile aligner can be considered to have a hydrophobic character and the Spark a hydrophilic character. These differences also translate into surface energies where these two aligners also present statistically significant differences [38].

It is interesting to note how the Spark aligner, unlike the other two—Invisalign and Smile—the contribution of the polar character in the surface free energy is very high with respect to the dispersive and therefore this aligner will have a tendency to other polar elements such as certain bacteria. These differences may be due to the fact that the chemical composition of the spark aligner contains trifluoro(methanol)boron, which gives it a polar character [39].

It is known that moderate hydrophilicity with angles around 90° produces the highest levels of bacterial colonization. Superhydrophilic surfaces have very little adsorption of bacteria because the surface adsorbs water very quickly preventing bacteria from coming in contact with the surfaces [40,41,42]. The same is true for superhydrophobic surfaces where the surface in this case is surrounded by air and bacteria that are in the physiological medium cannot adsorb on the surface [43]. We observed in our results that the Smile aligner is the aligner that presents contact angle values closest to 90° and is the one that gives the highest colonization values for *S. oralis*, *A. viscous* and *P. gingivalis* with statistically significant differences with respect to the Spark aligner. However, this aligner behaves in the opposite way with the bacterial strains *S. gordonii* and *E. faecalis*, where the aligner with a greater hydrophilic character has a greater colonization of these bacteria. This fact is due to the fact that these bacteria are anaerobic and their structure allows polar interactions at the membrane level. *E. faecalis* bacteria have high reactivity at the membrane level and produce reactions that generate glucose without gassing or react with catalases [44,45,46]. The other *S. gordonii* bacteria have a high avidity for other molecules given their high polarity and can even affect DNA sequences. These bacteria have a high reactivity in polar environments and as we can see from the results in Table 1, the Spark aligner has a large contribution to the surface free energy of the polar component [47,48,49,50].

There are authors who observe a change in the microbiota of dental surfaces as well as saliva after just 12 h of wearing aligners [15,34,48,49]. But others such as Zhao observed that the microbiota does not change significantly months after starting treatment with Invisalign aligners [51]. As our study is in vitro, without collecting saliva from patients or used aligners, we can assess that the surfaces of the various aligners have the capacity to be cultured and can be a good reservoir of these [40]. Yan et al. has observed that the microbiota of dental surfaces and saliva changes after wearing aligners in the mouth for 12 h [50,51,52,53,54].

Low, in his study, was able to observe bacterial colonization according to the time elapsed after wearing the aligners. After 6 h, he saw species of cocci that colonized the surfaces of the device. After 24 h, the colonization increased with a large mass of coccoid species dispersed in a thick extracellular matrix. After 48 h, filamentous and rod-shaped species already existed [52].

Therefore, this appliance would have the same capacity to retain bacteria as any other removable orthodontic appliance, or even more depending on the hours of use.

In Lombardo’s 2017 study [54], the amount of biofilm was measured using scanning electron microscopy. They use F22 aligners used after 14 days for their microbiological measurement. They cut a 6 × 6 mm area of each aligner from the vestibular surface of the upper right first premolar area, which they put in glutaraldehyde. They will then be evaluated with a scanning electron microscope magnified 10 thousand times. These will be measured using a gray scale for statistical evaluation. Although they observe the plaque formed, they do not distinguish strains for their study to measure plaque reduction with different cleaning agents [54].

Other research [55] observed that when measuring plaque at the start of treatment in patients switched to aligners and after the start of treatment, 13.6% of patients began to have Gram-negative pathogenic bacteria, observed under a microscope as motile, composed mostly of spirochetes (including *Treponema denticola*) and *Trichomonas tenax*. In the Moradinezhad study the microorganisms were cultured on 270 disks for 24 h (90 disks), 72 h (90 additional disks) and 5 days or 120 h (90 additional disks). Biofilm formation of the strains: *Streptococcus mutans*, *Streptococcus sanguini*, *Staphylococcus epidermidis*, *Staphylococcus aureus* and *Lactobacillus casei.* In addition to *Candida albicans*. He explains that a greater colonization by *Candida* was observed, more than by any bacteria, for the invisible retainers measured [56].

After measuring bacterial strains in orthodontic aligners, where they see quite a few strains, Sfondrini, explains that there are no statistically significant results in periodontal and microbiological parameters when compared with patients not treated during the first two months of therapy [57]. Mummolo observed after 6 months using aligners, 10% of patients with clear aligners and 13.3% of patients with removable positioners are at risk of developing caries due to colonization of the aligners by *Streptococcus mutans*. Therefore, it would be interesting for future studies to also evaluate colonization by *S. mutans* [58].

In the Pasaoglu study, six orthodontic clear aligner systems were selected to evaluate their bacterial colonization: Invisalign, Clarity, ClearCorrect, Smartee, Orthero and Graphy. In vitro strains of *Streptococcus mutans* (SM) (ATCC 25175) and *Lactobacillus acidophilus* (LM) (ATCC 4356) were used. When comparing the SM biofilm formation on different orthodontic aligners during the observation period from 0 to 240 h, in the tests performed for the ClearCorrect, Smartee, Orthero, Clarity, Graphy and Invisalign materials, statistically significant differences were detected between the measurements obtained at all times, except in the first 24 h of colonization. In all materials, the averages of the measurements increase as time progresses. This study provides valuable information on biofilm formation and microbial adhesion in different clear aligner systems. However, it is clear that the longer the time, the more formation, with long-term colonization being a concern, so these results should make us think about the importance of not prolonging the use of each aligner for too long, knowing that the choice of material and the design of the aligner can influence the oral microenvironment and bacterial adhesion [59].

Finally, in the study by Sifakakis, bacterial colonization of *S. mutans*, *L. acidophilus*, and *S. sanguinis* was evaluated in a sample of clear polyethylene glycol-terephthalate copolyester (PET-G) orthodontic aligners and self-ligating brackets. No differences were found in salivary levels of *S. mutans* and *L. acidophilus* between adolescent patients treated for 1 month; but lower salivary levels of *S. sanguinis* were seen in patients treated with thermoplastic aligners compared to those treated with self-ligating fixed appliances [60].

In relatio to the cytotoxicity, various in vitro studies such as those by Eliades [61] and Premaraj [62] have evaluated the compatibility of Invisalign aligners with gingival epithelial cells. Premaraj studied, in vitro, the biocompatibility of one of the components of the brand’s aligners, isocyanate, testing its cytotoxicity in epithelial keratinocytes, coinciding with another author, Hamada demonstrated that this component could cause allergic reactions on contact [63]. This is a line of research that should be evaluated if there were more aligners on the market with this component. But it is true that commercial companies do not reveal the proportions of the components of their aligners, making their evaluation difficult.

The cytotoxicity measurements were performed during 288 h of culture on human periodontal ligament cells with quaternary ammonium moiety-modified gold nanocluster aligners. These results demonstrate that the antibacterial coating on the surface of the aligners shows good biocompatibility with normal oral cells. But this study has no control group to evaluate cytotoxicity with Invisalign aligners without bactericide [64]. Martina’s contribution evaluates the cytotoxicity of four types of aligners made of polyethylene terephthalate glycol (PETG), polyurethane resin and multilayer polyurethane/copolyester aromatic thermoplastic materials. The material was exposed for 14 days. In vitro, all materials showed a slight cytotoxic effect on the last day, day 14, with comparable levels of cell viability, being higher for the PETG material. In our case, a greater difference was observed in the aligners made of Polypropylene/ethylene copolymer [65].

The cytotoxicity of aligners printed in 3D directly with light-curing polyurethane resin (Tera Harz TC-85, Graphy Inc., Seoul, Republic of Korea) was evaluated, but no differences were observed between the viability of exposed human gingival fibroblasts and controls. As in the Kim study, the toxicity of Tera Harz 3D printing resins is measured, which are highly cytotoxic before the 3D printing process and the cytotoxic levels are significantly reduced after removing the uncured resin by all post-polymerization procedures, being finally biocompatible [66,67].

In the Alhendi study [30] three samples of four commercial brands of orthodontic aligners were evaluated: Invisalign, Eon, Clarity and SureSmile. Invisalign and SureSmile demonstrated mild cytotoxicity with different concentrations of a 5% and 10% 3-[4,5-dimethylthiazol-2-yl]-2,5-diphenyltetrazolium bromide (MTT) solution, while they were moderate with concentrations of 20%. Clarity was mildly cytotoxic at all solution concentrations. For Eon aligners, mild cytotoxicity was seen with 5% and 20% solutions, but greater toxicity was recorded in the 10% solution, being moderate. Different materials were evaluated for the release of bisphenol A. Using severe mechanical and thermal conditions, BPA was observed to leach from the thermoformed Biocryl acrylic resin retention material and fully cured Transbond XT orthodontic adhesive within 3 days after immersion in artificial saliva. These were the only materials in the study with detectable amounts of BPA. Although bisphenol A was not a measurement in our study, given that this type of component is not safe for health, its contact with humans should be reduced [30].

The results have shown that the hypothesis is false and the positive hypothesis is confirmed based on the experimental results obtained. This research study has some limitations that should be taken into account. One of them is that human saliva should be used and the tests should be carried out at 37 °C with all the bacteria present in the mouth. Although the biofilm attempts to simulate what occurs in the mouth, this is an in vitro study and not an in vivo study, which means that it is an estimate of reality. It will be necessary to study the mechanisms of this wettability-surface free energy relationship and its relationship with aerobic and anaerobic bacteria, which is still highly controversial, and further testing will be required to clarify the dependency. However, the results show clear trends and, despite the limitations, the conclusions are clear and confirmed by the statistical studies carried out.

It should be noted that the aligners presented in the study are commercial products and have passed the regulations and quality tests of accrediting agencies as biomaterials. However, this study aims to gain a better understanding of aligners in order to optimize their performance in patients’ mouths. The results will enable improvements in wettability and surface free energy in different aligner materials to prevent the most pathogenic strains. This opens up research into the biofunctionalization of materials with peptides or other molecules that modify the physical chemistry of the surface or even have bactericidal properties, such as lactoferrin [68,69]. Also, since these are porous materials, substances with beneficial effects for the mouth could be included and released in a controlled manner. One example could be fluoride salts, which, when dissolved, could fight tooth decay due to the formation of fluorapatite, a much more resistant biomineral. However, care must be taken because if the patient has titanium dental implants, it could chemically attack the titanium, causing it to degrade.

## 5. Conclusions

The Spark aligners demonstrated lower metabolic activity against aerobic strains, possibly due to their hydrophilic character. However, they exhibited greater adhesion by anaerobic strains, likely associated with the polar component of surface free energy. All aligners showed cytocompatibility greater than 70%. These findings reinforce the importance of considering the physicochemical properties of aligner materials in clinical decision.

## Figures and Tables

**Table 1:** Wettability properties of the three aligners evaluated. CA, contact angle; SFE, total surface free energy; DIS, dispersive component; POL, polar component. The sum of the dispersive and polar components corresponds to the total surface free energy. An asterisk (*) indicates a statistically significant difference compared with values without an asterisk. A double asterisk (**) indicates a statistically significant difference compared with values without an asterisk and those with a single asterisk. Statistically significant differences were considered at *p* < 0.05.

	CA (°)	SFE (mJ/m^2^)	DISP (mJ/m^2^)	POL (mJ/m^2^)
Spark	70.5 ± 7.2	60.8 ± 9.8	28.9 ± 3.3	31.9 ± 3.4
Invisalign	80.6 ± 5.1 *	66.7 ± 1.5	47.4 ± 0.2 *	19,3 ± 1.5 *
Smile	91.2 ± 6.6 **	74.2 ± 2.1 *	50.2 ± 1.1 **	20.0 ± 2.2 *

**Table 2:** Metabolic activity of the different aligners studied with statistical analysis. (SD: standard deviation, ns: not significance).

Bacteria	Group	Mean	SD	Spark	Invisalign	SureSmile
*S. oralis*	Control	100	2.70473745	<0.0001	<0.0001	<0.0001
*S. oralis*	Spark	23.1467288	0.11968374	ns	<0.0001	
*S. oralis*	Invisalign	28.6743516	0.34232693			<0.05
*S. oralis*	SureSmile	38.5737936	0.15635539			
*A. viscosus*	Control	100	10.4870816	<0.0001	<0.0001	<0.0001
*A. viscosus*	Spark	43.1797115	0.53193343	ns	<0.05	
*A. viscosus*	Invisalign	52.1486041	1.4706573	ns		
*A. viscosus*	SureSmile	55.6004598	1.13615019			
*S. gordonii*	Control	100	4.11583083	<0.0001	<0.0001	<0.0001
*S. gordonii*	Spark	57.3586457	2.45147211	<0.0001	<0.0001	
*S. gordonii*	Invisalign	43.5841447	2.42727023		<0.0001	
*S. gordonii*	SureSmile	23.6468261	0.05131396			
*P. gingivalis*	Control	100	3.69664728	<0.0001	ns	<0.01
*P. gingivalis*	Spark	17.7484069	0.22514496	<0.0001	<0.0001	
*P. gingivalis*	Invisalign	78.1921171	15.4480032		ns	
*P. gingivalis*	SureSmile	71.245378	1.1596853			
*E. faecalis*	Control	100	1.21932032	<0.0001	<0.0001	<0.0001
*E. faecalis*	Spark	76.8894807	3.63631184	ns	<0.0001	
*E. faecalis*	Invisalign	76.0612517	3.70975383		ns	<0.0001
*E. faecalis*	SureSmile	51.5739015	0.27633597			
*Biofilm*	Control	100	0.79243285	<0.0001	<0.0001	<0.0001
*Biofilm*	Spark	2.43285861	0.06415541	<0.0001	<0.0001	
*Biofilm*	Invisalign	27.5373564	0.1068084		ns	
*Biofilm*	SureSmile	30.137912	0.20499682			

**Table 3:** Statistical results for the determination of CFUs. (SD: standard deviation, ns: not significance).

**Bacteria**	**Group**	**Mean**	**SD**	**Spark**	**Invisalign**	**SureSmile**
*S. oralis*	Control	15,000,000	0	ns	ns	ns
*S. oralis*	Spark	5	0			
*S. oralis*	Invisalign	44	1.414213562			
*S. oralis*	SureSmile	10,650,000	1,909,188.309			
*A. viscosus*	Control	7,808,000	2,375,878.785	<0.001	<0.01	<0.01
*A. viscosus*	Spark	615,000,000	445,477,272.1	ns	ns	ns
*A. viscosus*	Invisalign	180,000,000	127,279,220.6	ns	ns	ns
*A. viscosus*	SureSmile	1,140,000,000	933,380,951.2	ns	ns	ns
*S. gordonii*	Control	8,040,000	3,563,818.177	<0.01	<0.0001	<0.001
*S. gordonii*	Spark	1,845,000	784,888,527.1			
*S. gordonii*	Invisalign	450,000	212,132.0344			
*S. gordonii*	SureSmile	1,530,000	275,771,644.7			
*P. gingivalis*	Control	5,280,000	3,394,112,550	<0.05	<0.05	ns
*P. gingivalis*	Spark	285,000,000	21,213,203.44			
*P. gingivalis*	Invisalign	119,000,000	84,852,813.74			
*P. gingivalis*	SureSmile	840,000,000	254,558,441.2			
*E. faecalis*	Control	27,000,000	0	<0.0001	<0.0001	<0.0001
*E. faecalis*	Spark	300,000,000	127,279,220.6			
*E. faecalis*	Invisalign	315,000,000	190,918,830.9			
*E. faecalis*	SureSmile	150,000,000	21,213,203.44			
*Biofilm*	Control	4,740,000	933,380,951.2	ns	ns	ns
*Biofilm*	Spark	300,000,000	42,246,406.87	ns	ns	ns
*Biofilm*	Invisalign	480,000,000	84,852,813.74	ns	ns	ns
*Biofilm*	SureSmile	1,470,000,000	42,246,406.87	ns	ns	ns

**Table 4:** Statistical study of the cell viability.

Group	Mean	SD	Spark	Invisalign	SureSmile
**Control**	100	2.028959866	0.0083 *	0.1002	0.0040 *
**Spark**	70.88959285	9.749625257	—	0.3322	0.9348
**Invisalign**	75.71665974	12.87686935	—	—	0.1544
**SureSmile**	75.64914832	7.425628458	—	—	—

* Significant differences (*p* value < 0.01).

## Data Availability

The data presented in this study are available on request from the corresponding author. The data are not publicly available due to complexity of interpretation.
